# Artificial intelligence in diabetes care: from predictive analytics to generative AI and implementation challenges

**DOI:** 10.3389/fendo.2025.1620132

**Published:** 2025-11-19

**Authors:** Mengqi Deng, Ruiye Yang, Xiaoran Zheng, Yaoqi Deng, Junyi Jiang

**Affiliations:** 1 Department of Gynecological Oncology, Beijing Obstetrics and Gynecology Hospital, Capital Medical University, Beijing Maternal and Child Health Care Hospital, Beijing, China; 2 Department of Gynecology and Obstetrics, Handan Fukang Hospital, Handan, Hebei, China; 3 Department of Educational Management, Nanchang University, Nanchang, Jiangxi, China; 4 State Key Laboratory of Medical Proteomics, National Center for Protein Sciences, Institute of Lifeomics, Beijing, China

**Keywords:** generative artificial intelligence, public health informatics, medical AI governance, algorithmic fairness, explainable AI, data colonialism, health equity, ethical machine learning

## Abstract

Generative artificial intelligence (GenAI) is transforming public health and medicine as well, in the form of disease surveillance, resource allocation and clinical decision making. Interventions to improve efficiency — multimodal predictive algorithms, federated learning platforms — reveal the internal contradictions of the system between algorithmic efficiency and fairness: speed of technical innovation and regulatory deficit, data flows without borders vs. ethical values of places. We present a three-dimensional governance structure for the topic covering the technical, institutional and ethical domains. From a technology point of view, explainability solutions and culturally-aware design align transparency with cultural sensibility. From an institution point of view, privacy-protecting data platforms and risk-based regulation align innovation with accountability. From an ethical point of view, incorporating local values and disbursing AI dividends sustain equitable health outcomes. There are still challenges that demand the utmost priority, including the algorithmic prejudice, the data imperialism and the opacity in medical AI decision making. Future priorities include the development of broader measurement tools that integrate clinical impact, equity, and societal impact; the development of transnational governance institutions to mitigate concerns relating to data sovereignty; and the development of forms of participatory design between designers, practitioners, and populations. A balance between technical creativity, visionary policy-making, and caring leadership to advocate for human-centered healthcare will provide us with trusted AI ecosystems. Technical excellence alone cannot guarantee success unless fairness and accessibility, social responsiveness, and justice for future global health is guaranteed.

## 1 Introduction

The global diabetes epidemic has reached substantial proportions, with an estimated 537 million adults currently affected, a number projected to rise significantly within the next decade (1). This trend is mirrored in China, where national surveys indicate a dramatic increase in prevalence from over 1% in 1980 to over 10% in 2017, with current data suggesting approximately 13% of the adult population lives with diabetes (2). The associated morbidity, mortality, and economic burden are profound; in 2019, diabetes-related causes accounted for 0.82 million deaths among Chinese adults and represented a leading source of healthcare expenditure (2). Coupled with the disability resulting from its complications, diabetes stands as one of the most critical public health challenges of the 21st century (3, 4). Traditional diabetes management faces several challenges, including under-diagnosis, suboptimal treatment, and the resource-intensive nature of aggressive care, which requires coordinated efforts from endocrinologists, nutritionists, nephrologists, ophthalmologists, and other specialists—resources often scarce or unevenly distributed (2). Furthermore, achieving optimal glycemic control remains difficult as it heavily depends on patient behaviors such as dietary intake, physical activity, and glucose monitoring (5).

Digital health technologies, especially Artificial Intelligence(AI), hold immense potential to address these gaps (6). While numerous reviews have cataloged AI applications in diabetes care, they often remain siloed within technical domains (e.g., prediction, diagnosis) (**Table 1**). This review advances the discourse by introducing a patient-centric “IPAES” framework (Identification, Prediction, Assistance, Education, and Support) that maps AI technologies to the complete patient journey, while critically examining the real-world implementation barriers—algorithmic fairness, clinician trust, regulatory hurdles—that determine ultimate translational success (1, 4). We pay particular attention to the emerging role of generative AI, which moves beyond traditional discriminative models to create novel content and solutions, and we explore its potential to revolutionize areas like personalized patient education and synthetic data generation (3, 7).

**Table 1:** Key characteristics of AI applications in diabetes management.

Application domain	Typical AI technologies/tools	Key features
Prediction & Prevention	Machine learning-based risk prediction models	Utilize multidimensional data mining to identify high-risk populations, enabling early intervention
Screening & Diagnosis	Non-invasive imaging classification (retinal photo recognition), Diagnostic assistance systems	Automated screening/diagnosis, non-invasive approach, high accuracy, improved identification of high-risk individuals
Integrated Management	Intelligent health education systems, Diet-exercise recommendations, CGM prediction algorithms, Insulin dosage optimization	Personalized nutrition/exercise guidance, real-time glucose monitoring & alerts, automatic insulin adjustment, enhanced patient self-management
Complication Management	Deep learning-based DR screening, CKD risk prediction, Wound/foot recognition, Neuropathy screening	Multimodal data analysis enables early complication detection and risk assessment, prevents severe complications

AI can parse vast amounts of multimodal health data—including electronic health records, genomics, medical images, and data from wearables—to assist both clinicians and patients (2, 6). Recent reviews highlight AI applications across the entire spectrum of diabetes care, from enhanced screening and diagnosis to treatment management and complication prediction (3, 4). In risk prediction, for instance, algorithms using clinical and biological features can accurately identify individuals at high risk for type 2 diabetes (4). Multimodal models integrating genomic, metabolomic, and clinical data have demonstrated exceptional performance, with one study reporting an area under the receiver operating characteristic curve (AUC) of approximately 0.96 (6). Deep learning models applied to ophthalmic imaging have also shown remarkable accuracy; convolutional neural networks interpreting retinal fundus photographs and clinical metadata have achieved AUROCs between 0.85 and 0.93 for detecting prevalent type 2 diabetes (6).

AI has also significantly advanced diabetes classification and treatment personalization. While diabetes has traditionally been categorized primarily into type 1 and type 2 (with additional categories such as gestational diabetes), data-driven clustering analyses suggest a more nuanced subtyping (8, 9). Ahlqvist et al.’s groundbreaking study, using six clinical variables, identified five reproducible clusters of adult-onset diabetes with distinct phenotypes and complication risks (8). These subgroups have been replicated across diverse populations (9), suggesting improved phenotyping that could enable precision management. Moving beyond subclassification, AI is now directly informing therapeutic choices. A landmark study by Dennis et al. (2025) developed and validated a predictive model using routinely available clinical features to compare the efficacy of five major drug classes for type 2 diabetes, providing a data-driven tool to optimize individual patient prescribing at diagnosis (10).

Furthermore, AI is transforming patient self-management and education. Mobile health (mHealth) interventions integrating AI-powered virtual health assistants can improve medication adherence and glycemic outcomes (9, 11). For example, a pilot study demonstrated that an intelligent mobile self-management system for type 2 diabetes effectively reduced HbA1c (11). A separate 12-week trial showed that an AI-guided smartphone educational program led to significantly better glycemic control compared to standard care (12). Natural language processing (NLP) has been utilized to analyze patient forum discussions and generate customized educational content (13). AI also contributes to nutrition management; image-based systems can estimate dietary intake from meal photos, mitigating the inherent biases of self-reported food diaries (5, 14). Additionally, advanced AI-driven insulin dosing algorithms can help clinicians evaluate continuous glucose sensor data and recommend adjustments to basal insulin regimens, thereby improving glucose levels (4). However, the adoption of these recommendations hinges on effective clinician-AI collaboration; providers must be equipped to interpret the algorithm’s rationale and reconcile it with their clinical judgment and patient preferences (15, 16).

Generative AI, particularly large language models (LLMs), represents a paradigm shift beyond traditional predictive analytics (3, 7). These models can parse and generate human-like text, potentially integrating patient information, medical literature, and behavioral feedback to create highly personalized educational content, simulate patient interactions for clinician training, and even generate synthetic datasets to augment limited real-world data while preserving privacy (3). The promise of highly customized care persists, even amid challenges related to algorithmic bias, data privacy, and clinical validation (1, 4).

This review provides an overview of recent advances through the IPAES lens, focusing on AI-driven predictive modeling, screening, classification, and therapy optimization to evaluate its current status in diabetes management. It explores how these tools may make diabetes care more precise, pervasive, predictive, and personalized. While discussing AI’s potential to improve outcomes, we also address the critical challenges of data quality, algorithm design, fairness, and clinical adoption that must be overcome for its full realization (1, 4).

## 2 Digital and telemedicine-enabled care, augmented by AI

**Figure 1:**
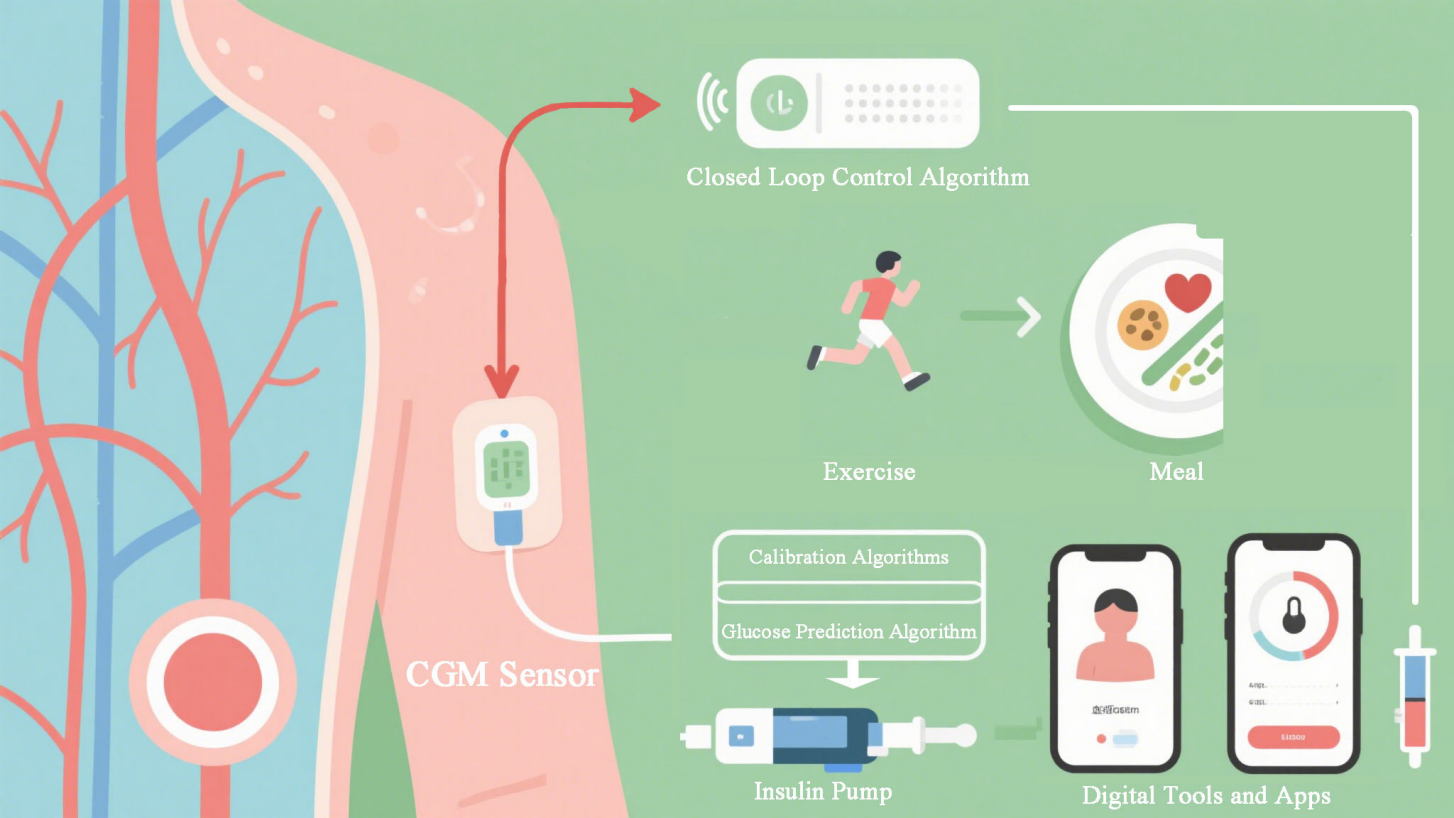
AI algorithms in diabetes management.

Digital health tools, including internet-based platforms, mobile applications, wearables, and telemedicine, are becoming integral to modern diabetes management (1, 17) (**Figure 1**). Telemedicine allows clinicians to remotely provide dietary and chronic disease management, overcoming geographical and resource barriers (17, 18). AI is a key enabler within these digital tools, powering the analytics and personalization that make them effective (2, 4).

Recent trials have shown that home-based telemedicine programs integrating exercise training and personalized nutritional counseling can lead to modest but significant improvements in glycemic control for individuals with type 2 diabetes and coronary heart disease (18). A large-scale pragmatic trial in Brazil demonstrated that diabetes-related teleconsultations were non-inferior to face-to-face consultations in reducing HbA1c (17), validating the transformative potential of telemedicine. Mobile and web-based tools further extend tele-enabled care by automating diet and nutrition monitoring. AI-powered applications like GoCARB and Snap-n-Eat use image analysis to estimate carbohydrate and calorie content of meals with accuracy comparable to dietitians, thereby avoiding the well-documented problems of self-reported food intake (5, 14). A critical question for real-world implementation is whether diverse patient populations will trust and adhere to AI-generated dietary advice, which may not always align with cultural preferences or socio-economic constraints, highlighting the need for culturally adaptive algorithms and shared decision-making (19, 20).

The impact of AI-enhanced, patient-tailored mHealth interventions is significant. For instance, an intelligent mobile diabetes education system led to reduced HbA1c and improved patient knowledge (11, 12). Meta-analyses consistently report that app-based interventions improve glycemic control; a recent review of 41 randomized controlled trials (RCTs) found that diabetes self-management apps reduced HbA1c by approximately 0.5% compared to routine care (21). Benefits extend beyond glucose metrics; a meta-analysis of tele-nutrition trials in patients with cardiovascular disease showed slight but significant reductions in systolic blood pressure and LDL-cholesterol (22). Systematic reviews confirm that digital interventions (including telemedicine, SMS, and web programs) can reduce weight and improve glucose parameters in at-risk populations (23).

Telemedicine is complemented by wearable sensors and remote monitoring, which provide continuous data to healthcare providers (24, 25). AI algorithms are central to interpreting this data stream, enabling tight glucose control through remote coaching and automated feedback (4, 26). Home-based studies of AI-driven, automatically adjusted insulin-dosing algorithms and closed-loop systems have demonstrated improved glycemic outcomes (21, 26). When combined, these digital tools facilitate more individualized nutrition and chronic disease care. Telemedicine platforms provide access to dietitians and self-management training, while AI applications offer objective dietary assessment and automated decision support (14, 21). This integrated digital nutrition care has the potential to improve clinical outcomes. However, equitable access remains crucial, as digital technologies could potentially exacerbate health disparities if not implemented thoughtfully (19).

## 3 The generative AI revolution in diabetes management

While discriminative AI models (e.g., for prediction and classification) have been the focus of most prior reviews, generative AI offers a suite of novel capabilities that promise to reshape diabetes care (3, 7). Unlike discriminative models that predict an output from an input, generative models create new data—text, images, or even synthetic patient records—that resemble real-world data (7). This capability unlocks unique applications.

### 3.1 Personalized patient education and communication

Generative AI, particularly large language models (LLMs), can produce tailored educational materials, answer patient queries in real-time, and simulate empathetic conversations (20, 27). In a similar fashion, a generative AI assistant could explain complex glycemic concepts in a culturally and linguistically appropriate manner, adapting its explanations based on a patient’s literacy level and personal context (7, 20). This moves far beyond static app content or templated messages, enabling dynamic, interactive patient support that can improve health literacy and engagement (11, 20).

### 3.2 Synthetic data generation

A significant barrier to robust AI development in diabetes is the scarcity of large, diverse, and well-annotated datasets, often due to privacy concerns (6, 28). Generative AI can create high-fidelity, synthetic patient data that mirrors the statistical properties of real data without containing any identifiable personal information (7). This synthetic data can be used to train more robust and generalizable machine learning models for tasks like risk prediction, to test clinical decision support systems, and to augment datasets for rare diabetes subtypes, thereby mitigating biases inherent in small, homogenous datasets (7, 28).

### 3.3 Clinical workflow augmentation

Generative models can assist clinicians by drafting clinical notes from verbal patient encounters, generating summary reports from complex patient data (e.g., from CGM, EHRs), and even suggesting differential diagnoses or personalized care plan drafts (7). This can reduce administrative burden, allowing clinicians to focus more on direct patient care (29). The integration of image-based deep learning with language models is a particularly powerful trend. Li et al. (2024) demonstrated an integrated system for primary diabetes care that combines the analysis of retinal fundus images with clinical data processing via language models, showcasing a multimodal AI approach that can support comprehensive patient assessment and management planning at the primary care level (7).

The integration of generative AI into diabetes management is still nascent, and challenges regarding factual accuracy (“hallucinations”), safety, and ethical oversight are paramount (3, 7). However, its potential to move beyond analysis to creation positions it as a transformative tool for personalization and scalability in diabetes education and support (7, 15).

## 4 Algorithmic fairness and health equity in AI for diabetes

While “Algorithmic Fairness” and “Health Equity” are critical keywords, they demand substantive discussion. The performance and safety of AI models are not uniform across populations, and without deliberate effort, these technologies can perpetuate or even exacerbate existing health disparities (19, 28).

### 4.1 Performance disparities

AI models trained on datasets from high-income, Western populations may perform poorly when deployed in other settings (28). For example, a retinopathy detection algorithm trained primarily on retinal images from Caucasian populations may have reduced sensitivity when applied to patients of different ethnicities due to variations in fundus pigmentation (6, 28). Similarly, risk prediction models using genetic data are often biased if the training data lacks diversity, as genetic markers for diabetes can vary across ancestries (30). This risk of “data colonialism,” where models built on data from well-represented groups are deployed without validation in under-represented populations, is a major ethical concern (28). Performance gaps have been observed along socioeconomic lines as well; models relying on smartphone or wearable data may be inherently biased against underserved populations who have lower access to these technologies (19, 25).

### 4.2 Equitable implementation and non-invasive diagnostics

Beyond algorithmic bias, equitable implementation is a key challenge (19). AI-driven solutions, such as smartphone-based retinopathy screening, hold particular promise for low-resource settings (e.g., rural India) where specialist access is limited (6, 28). These tools can decentralize screening and improve early detection (6). Furthermore, AI is enabling novel, less invasive diagnostic pathways for complications. A pioneering study by Meng et al. (2025) demonstrated that a deep learning model applied to retinal images could non-invasively biopsy and diagnose diabetic kidney disease, offering a potentially more accessible and scalable screening tool compared to repeated urine and blood tests, which is particularly relevant for underserved areas (28). However, their success depends on addressing contextual barriers: digital literacy, language localization, connectivity issues, and integration into often-fragmented public health systems (19). Ensuring that AI tools are designed *for* and *with* low-resource settings, rather than simply being deployed there, is crucial for achieving equity (19, 28).

Safeguarding algorithmic fairness requires the implementation of a multi-faceted strategy (16, 28). A primary step involves the conscious curation of development datasets that are truly representative, encompassing the full spectrum of age, gender, ethnicity, socioeconomic status, and geographic location (28). Following this, rigorous robustness and fairness testing is indispensable, which entails evaluating models for performance disparities across demographic subgroups prior to deployment and instituting continuous monitoring for performance drift in real-world settings (16). Furthermore, the practice of independent algorithmic auditing should be established to systematically assess models for hidden biases (16). The adoption of technical approaches like federated learning also presents a significant opportunity, as this method enables model training across multiple institutions without the need to share raw patient data, thereby facilitating learning from diverse populations while simultaneously upholding privacy and complying with data residency laws, which in turn helps mitigate the risks of centralization bias (16, 28). Ultimately, it must be emphasized that addressing fairness and equity is not a peripheral consideration but a fundamental prerequisite for the responsible and effective global deployment of AI in diabetes care (19, 28).

## 5 Implementation challenges and future directions

The translation of promising AI innovations into routine clinical practice faces significant headwinds. A dedicated focus on these implementation science barriers is critical for moving from proof-of-concept to widespread impact (1, 4).

The widespread adoption of AI in diabetes care faces several multifaceted barriers that extend beyond technical performance (1, 4). A primary technical challenge lies in achieving seamless interoperability with Electronic Health Records (EHRs), as the integration of AI tools into existing clinical workflows requires smooth data exchange and minimal disruption to established practices; without this, even the most accurate algorithms will experience low clinician adoption (31).

Compounding these technical challenges are complex and evolving regulatory hurdles for AI-based Software as a Medical Device (SaMD) (4). Regulatory bodies are currently adapting to the unique demands of governing both “locked” static algorithms and “adaptive” continuously learning systems, which necessitate frameworks for ongoing monitoring and validation throughout their lifecycle (4, 15).

Underpinning all technical and regulatory considerations are the critical human factors of clinician trust and patient acceptability (15). The opaque “black box” nature of many complex models can significantly erode clinician confidence, necessitating new paradigms for effective collaboration where AI systems must provide not only recommendations but also contextual, explainable rationales and clear statements of their limitations (16). This enables clinicians to apply their expertise in evaluating AI-driven suggestions, such as insulin dosing recommendations (16, 26). Simultaneously, building basic AI literacy among healthcare professionals is essential for the critical evaluation and appropriate application of these tools (15). Furthermore, the success of AI interventions is equally dependent on patient trust and willingness to adopt AI-generated advice, such as dietary plans (20). Fostering this trust demands transparent communication about the role of AI in care, demonstrable accuracy, and system designs that incorporate user-centered feedback and cultural sensitivity, thereby engaging patients as active participants in their own management (15, 20).

The foundation of any effective AI system is high-quality data and robust infrastructure, yet significant obstacles persist due to issues like missing data, incorrect labels, and inconsistent data collection practices across different healthcare institutions, all of which can severely compromise model performance and generalizability (2, 6). Finally, the long-term sustainability of AI-augmented care is hampered by the current lack of clear reimbursement models (1). For health systems to sustainably invest in these technologies, it is imperative to demonstrate not only clinical efficacy but also compelling cost-effectiveness and a clear return on investment, proving the value of AI beyond mere technical innovation (1, 21).

Navigating a successful path forward necessitates a concerted and multi-faceted strategy that addresses the identified barriers holistically (1, 4). A foundational step involves the concerted development of common data standards and interoperability frameworks, which are crucial for enabling seamless integration of AI tools into diverse clinical ecosystems and ensuring that data can flow securely and efficiently between systems (31).

Concurrently, there must be a dedicated focus on advancing the field of Explainable AI (XAI), prioritizing the development and validation of techniques that move beyond theoretical transparency to provide clinicians with actionable, clinically meaningful insights that they can trust and utilize in their decision-making processes (16). The success of this technological advancement is inextricably linked to profound stakeholder engagement; this requires actively involving clinicians, patients, and healthcare administrators in the co-design of AI tools from the very outset, ensuring that the solutions developed are aligned with real-world workflows, patient needs, and organizational capabilities (15).

Finally, a strategic shift towards implementation science research is paramount, where scholarly inquiry expands beyond establishing algorithmic efficacy in controlled settings to rigorously studying and defining effective strategies for deploying, sustaining, and scaling these technologies across the vast and varied landscape of clinical practice (1). By proactively embracing this comprehensive approach, the global diabetes community can systematically dismantle the barriers to adoption and ensure that the tremendous potential of AI translates into tangible, equitable, and scalable improvements in patient care and outcomes (1, 4).

## 6 Conclusion

Advances in technology and therapeutics are reshaping diabetes management. Digital health tools—increasingly powered by both discriminative and generative AI—offer unprecedented opportunities to personalize and optimize care (1–4). This review has framed these advances through the IPAES framework, highlighting the journey from Identification to Support, while critically examining the frontiers of generative AI, algorithmic fairness, and implementation science (1, 4). Recent evidence, including high-impact studies on treatment optimization, non-invasive diagnostics, and integrated multimodal models, demonstrates that these innovations can improve glycemic control, patient satisfaction, and care efficiency (7, 10, 21, 28). Concurrently, the enduring importance of lifestyle modification and psychosocial support remains clear (32, 33). The future of diabetes care lies in integrating these elements into coherent, patient-centered care pathways, combining AI-enabled platforms with multidisciplinary teams (34, 35). Success, however, hinges on overcoming the critical barriers of interoperability, regulation, and—fundamentally—fostering trust and enabling effective collaboration between clinicians, patients, and intelligent systems (1, 15). As the global diabetes community moves forward, an emphasis on ethically deployed, holistic, and implementable innovations will be paramount to ensuring that technological advances translate into equitable, real-world health benefits for all populations affected by diabetes (1, 19).
